# Surgical tips and techniques to avoid complications of thyroid surgery

**DOI:** 10.1515/iss-2021-0038

**Published:** 2022-10-11

**Authors:** Christos K Stefanou, Georgios Papathanakos, Stefanos K Stefanou, Kostas Tepelenis, Aikaterini Kitsouli, Alexandra Barbouti, Periklis Tsoumanis, Panagiotis Kanavaros, Panagiotis Kitsoulis

**Affiliations:** Department of Surgery, General Hospital of Filiates, Thesprotia, Greece; Intensive Care Unit, University Hospital of Ioannina, Ioannina, Greece; Department of Endocrine Surgery, Henry Dunant Hospital Center, Athens, Greece; Department of Surgery, University Hospital of Ioannina, Ioannina, Greece; Department of Anatomy-Histology-Embryology, University of Ioannina, Ioannina, Greece; Department of Ophthalmology, University Hospital of Ioannina, Ioannina, Greece; Medical School, University of Ioannina, Ioannina, Greece

**Keywords:** complications, hemorrhage, hypoparathyroidism, recurrent laryngeal nerve, superior laryngeal nerve, thyroid surgery, thyrotoxic storm

## Abstract

**Objectives:**

Surgery of the thyroid takes place in a body part with complicated anatomy and several vital physiologic functions. Thyroidectomy is rarely associated with mortality but can be followed by significant complications, (i.e. hypoparathyroidism, hemorrhage, upper airway obstruction, laryngeal nerve injuries and thyrotoxic storm). This review aims to indicate surgical tips and techniques to sustain a low level of complications.

**Content:**

MEDLINE database (PubMed) platform was used as a search engine and the articles related to the topic were selected using the keywords combination “thyroid surgery and complications”.

**Summary and Outlook:**

The most common complication of total thyroidectomy with an occurrence ranging between 0.5 and 65% is hypoparathyroidism. Damage to recurrent laryngeal nerves can be temporary or permanent, unilateral or bilateral; bilateral lesion is associated with severe episodes of breathlessness. Thus, intraoperative monitoring of nerve function is essential to prevent damage. Ιn addition, hematoma formation can lead to breathing difficulties due to airway obstruction; preventive hemostasis during surgery is essential. The surgeon must have a complete anatomical understanding of not only the normal anatomy of the central visceral compartment of the neck, but also the common variations of the laryngeal nerves and parathyroid glands in order to keep the complication rate at a very low level.

## Introduction

Thyroid surgery, especially total thyroidectomy is one of the most commonly operations performed for endocrine pathology [1]. During the 1800s, the mortality from thyroid surgery was 40%, due to infection or haemorrhage [2]. At the 1900s Emil Theodor Kocher was the first who established the basic principles of thyroid surgery, which remained the same till our days and reduced the complications at a significant rate [3]. His basic principles constituted the thorough knowledge of the thyroid gland’s surgical anatomy, the gentle handling of the important structures and the good hemostasis.

The major complications during or after thyroidectomy are bleeding, injuries to the laryngeal nerves, hypoparathyroidism and thyrotoxic storm. The minor complications include postoperative seromas and poor scar performance. These complications are related to disease, the type of resection and the surgeon’s training and experience [4]. Better surgical experience is followed by fewer complications [5].

## Complications of thyroid surgery and how to avoid them

Nowadays, surgical principles improved surgical techniques and equipments have made complications from thyroid surgery rare.

### Minor complications

Minor complications are the most common in thyroid surgery, at a rate of 40% and include the following: Mild dysphagia, hoarseness, a degree of voice alteration, seromas and poor scar formation [6]. Postoperative seromas can occur and their treatment depends on the size and the symptoms; small size and asymptomatic one should be followed clinically until its full absorption. Large seroma can be initially aspirated under sterile conditions and clinically followed up thereafter; repeated aspirations may be necessary.

Non-aesthetic scars are another frequent complication. The incision should be placed on a natural crease of the neck and the length should be consistent with the thyroid gland’s measurements. The neck should be initially flexed so that it is possible to assess the position of the natural skin creases. The incision may be extended to the same skin crease if a lateral neck dissection is required. The wound should always be carefully treated, without unnecessary burns. If a cheloid scar occurs, the final cosmetic outcome may be improved by silicon compressive dressings or steroid injections.

### Major complications

Potential significant complications of thyroid surgery include hypoparathyroidism, haemorrhage, injury to the recurrent laryngeal nerve, injury to the superior laryngeal nerve and thyrotoxic storm.

#### Hypoparathyroidism

Hypoparathyroidism is the most common complication of thyroid surgery. The incidence of transient hypocalcemia is 27% and of permanent hypocalcemia is 1% [7]. Hypoparathyroidism occurs after injury, devascularization and inadvertent excision of the parathyroid glands [8].

**Figure 1:**
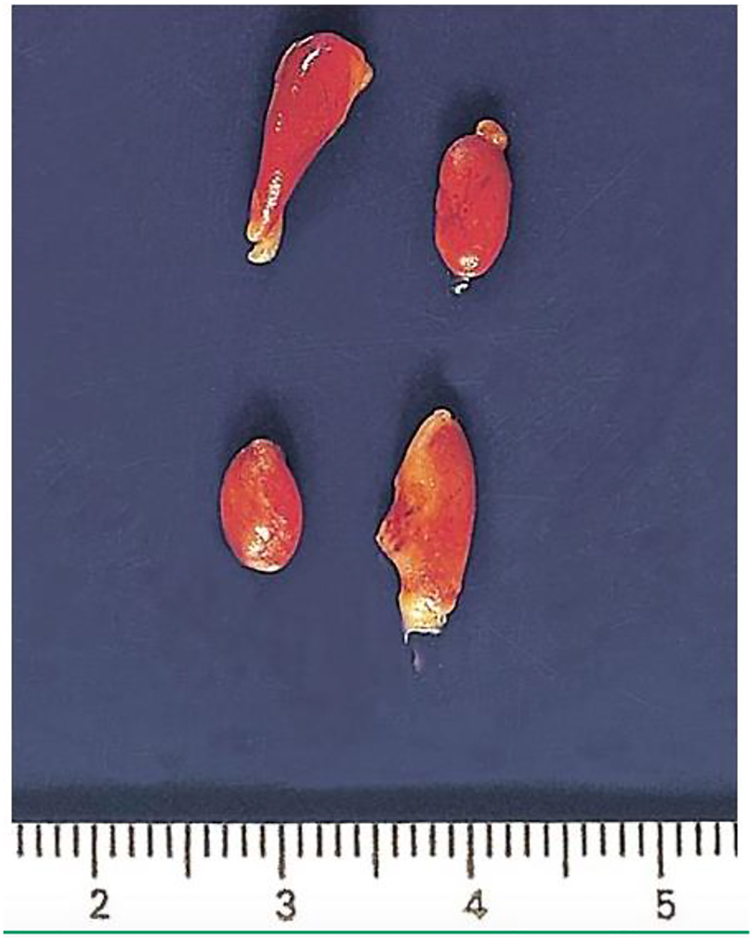
Four normal parathyroid glands.

The parathyroid glands are very tiny structures. Their dimension varies from 6 to 8 mm, their weight is between 20 and 50 mg and they are brownish in colour (Figure 1). Parathyroid hormone (PTH), which is involved in serum calcium regulation, is released by them. PTH increases serum calcium levels by causing bone resorption, increasing renal absorption of calcium and stimulating vitamin D synthesis [8].

In order to prevent their inadvertent excision or devascularization, understanding the potential anatomical variations of the parathyroid glands is very necessary. Typically, they are placed on the thyroid lobe’s superior lateral aspect. However, they can be located on any site from the hyoid bone down to the superior mediastinum, including an intra-thyroidal location. In 7% of patients, the probability of intra-thyroidal position occurs [9, 10]. Therefore, in order to locate any resected gland and to reimplant the gland in the cervical muscle, it is advised to open the surgical specimen of all thyroidectomies [11]. The superior parathyroid gland is usually posteriorly to the recurrent nerve. The inferior parathyroid gland, on the other hand, experiences a more variable situation and is commonly anterior to the recurrent nerve as it is close to the thyroid gland.

The parathyroid glands’ blood supply is very fragile. In the sub-capsular plane, the thyroid lobe must be dissected, avoiding mass ligatures of the inferior thyroid artery, which is normally the source of blood supply to both parathyroid glands. Ligation of the inferior thyroid artery terminal branches as close as possible to the thyroid gland decreases the risk of ischemia [12]. Their vascularization should be identified *in situ*, and they should be carefully manipulated. The viability of the parathyroid glands should be also tested always after the thyroidectomy; if they are identified as no more viable, it is preferable to re-implant them in a cervical muscle after mincing them. During thyroidectomy preserving at least one parathyroid gland with an adequate blood supply seems to prevent permanent hypoparathyroidism [13]. Surely, to prevent or minimize hypoparathyroidism after thyroidectomy, anatomical knowledge and surgical experience are important. The preferred method for the identification of iatrogenic hypoparathyroidism postoperatively is to measure the ionized calcium levels in the postoperative period. Alternatively, a normal postoperative PTH level can reliably predict normocalcemia after thyroid surgery. Acute hypocalcemia symptoms are perioral paresthesia, anxiety and tingling in the hands and legs [14]. Chvostek sign or the Trousseau sign are also indicators of hypocalcemia. Treatment of the hypocalcemia in the postoperative period depends on the symptoms. Patients with asymptomatic hypocalcemia should not be treated with supplemental calcium, because the hypocalcemia stimulates the stunned parathyroid glands to produce PTH. Otherwise, patients with symptomatic hypocalcemia should be treated with intravenous calcium gluconate [15].

The surgical technique is related to injury, oedema, infarction, ischemia or inadvertent excision of the parathyroid glands, but even if these glands are well preserved at the surgery, the normal postoperative parathyroid function is not guaranteed [16, 17]. Common risk factors for postoperative hypocalcemia are:

– Failure to recognize the parathyroid glands and inadvertently excise them [20].
– Devascularization: The final branch of blood supply to the parathyroid glands is mostly in the lateral to medial direction. Therefore, preservation of these vessels is essential for preventing devascularization.
– Intracapsular localization of the parathyroids (one or more) [9, 19].
– Diabetes mellitus: Parathyroids from diabetic patients are most vulnerable to hypoxia and delay to regain normal function due to diabetic microangiopathy [20].

#### Post-operative haemorrhage

**Figure 2:**
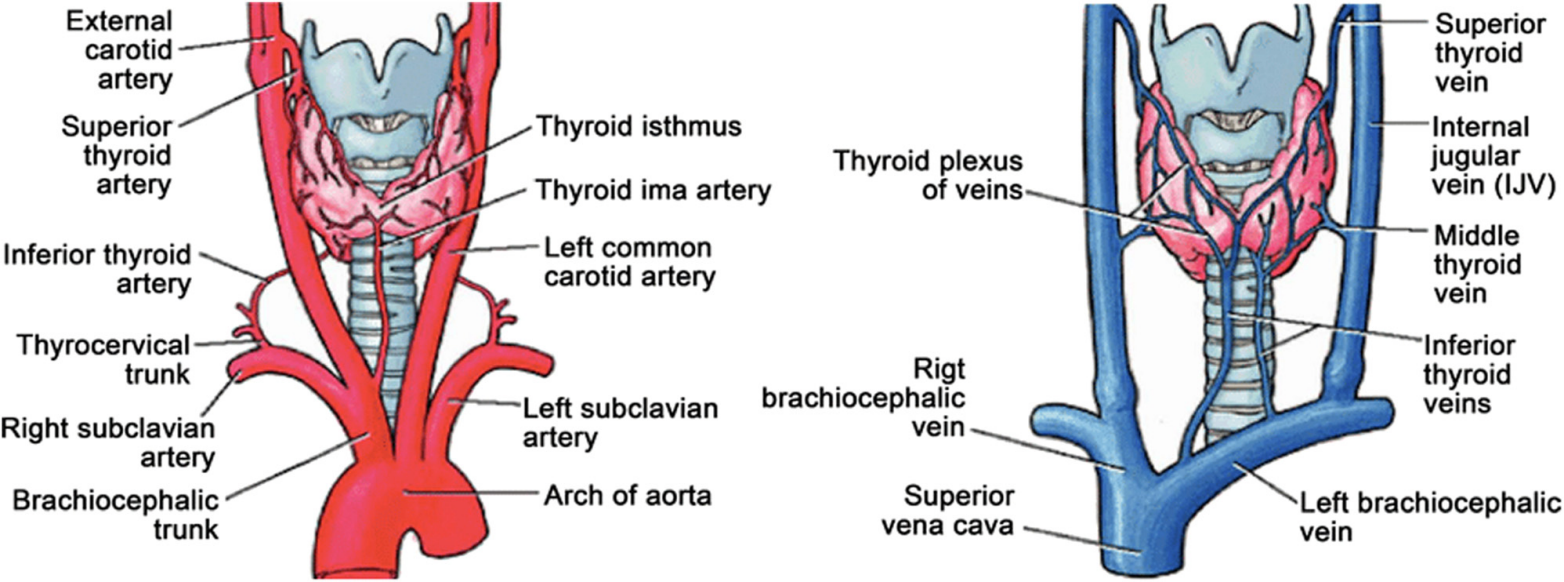
Arterial supply and venous drainage of the thyroid gland.

Post-operative haemorrhage can occur immediately in the post-operative period of thyroid surgery, with an incidence varying from 0 to 6.5% [21, 22]. The thyroid gland has a rich vascular supply, both arterial and venous, and hemostasis must be executed very carefully during the surgery (Figure 2). Risk factors for developing haemorrhage after thyroid surgery are hypertension, hyperthyroidism, bleeding disorders, duration of surgery and male gender [21, 22]. Thus, the appropriate pharmacological therapy must properly correct hypertension and hyperthyroidism before surgery [11]. Anticoagulants and antiplatelets increase hematoma incidence to 2.2–10.7%, which is significantly higher than the overall post-operative haemorrhage rate of 0.5%. Moreover, it has shown that injectable anticoagulants increase hematoma’s risk by 30 times [23]. According to all these, it is very important to evaluate the anticoagulant therapy and the thrombotic risk preoperatively.

Fortunately, the current use of ultrasonic or radiofrequency scalpels and the haemostats significantly reduces bleeding compared to older conventional technique with vessel ligations [24, 25]. The surgeon should check the open blood vessels, particularly the veins, with the Valsalva manoeuvre performed with the aid of the anaesthesiologist at the end of the surgery.

Drainage (gravity or suction-driven) can be placed according to surgeon’s preference. It must be emphasized that the drain does not prevent a serum or life-threatening hematoma from developing and raises the risk of wound infection and hospital stay [26, 27]. A post-operative hematoma creates an obstacle to venous and lymphatic flow, and also, if massive, results to an acute airway obstruction leading to asphyxia and death. Thus, if a post-operative hematoma is rapidly expanding, the surgical wound must be opened immediately at the bedside and if necessary even an emergency tracheostomy or cricothyroidostomy must be performed.

#### Recurrent laryngeal nerve injury

Thyroidectomy is a procedure during which the recurrent laryngeal nerve (RLN) identification and safety is very important. RLN identification minimizes the risk of injury to a rate of 0–2.1% and if not properly performed the risk increases to a rate of 4–6.6% [28].

The anatomical path of RLN differs between the right and the left side of the neck. The left RLN branches from the vagus at the aortic arch level, while the right RLN branches from the vagus more superiorly, at the level of the subclavian artery. Then the nerve courses towards the larynx on the tracheo-oesophageal groove, with a close anatomical relationship with the thyroid gland, parathyroid glands and the inferior thyroid artery [11]. The nerve enters the larynx through the Berry’s ligament, and serves as the main motor nerve of all the intrinsic laryngeal muscles, except the cricothyroid muscle.

The RLN has numerous pathways, sizes and calibers that increase the risk of complications and are vulnerable, especially in areas such as the Berry ligament, the Zuckerkandl tubercle, the tracheoesophageal groove, the inferior and superior thyroid poles, as well as during level VI dissections [29, 30]. The nerve can bifurcate or even trifurcate proximal to its laryngeal entrance; therefore, before the surgeon is 100% secure that a nerve branch is not inside the tissue involved, no part of the Berry’s ligament should be cut or clamped [31]. The ligament may wrap the nerve around it, so neuropraxia may be caused by excessive medial traction when finishing the dissection of the posterior part of the thyroid lobe. In addition, any attempt in this field to cauterize bleeding vessels can injure the nerve [11]. The distal part of the nerve is mainly protected by the tubercle of the Zuckerkandl (traverses medial to the tubercle and is shielded from view until the tubercle is retracted medially) or a portion of the Berry’s ligament or both of them [31, 32].

**Figure 3:**
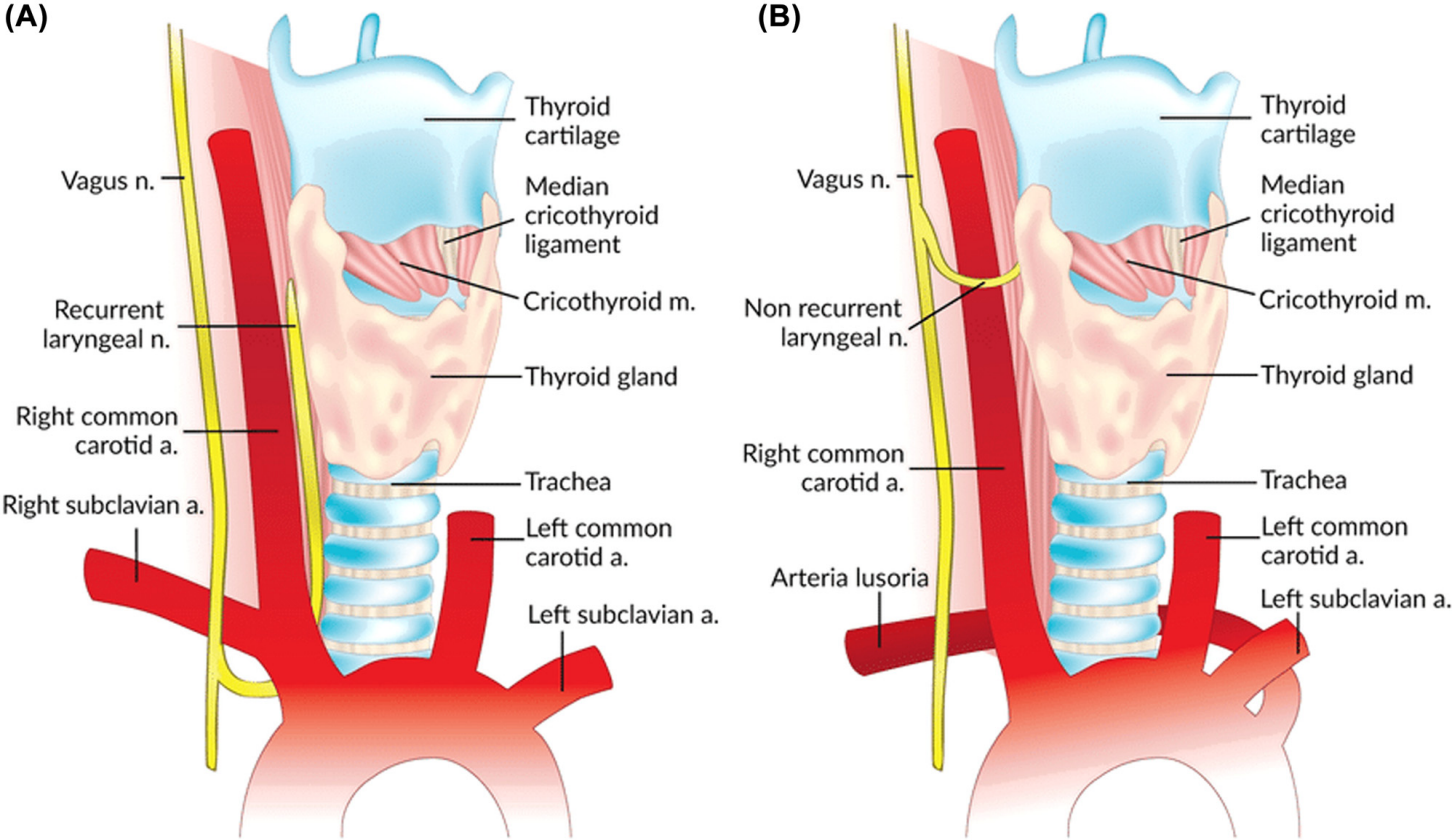
Normal right recurrent laryngeal nerve (A) and right nonrecurrent laryngeal nerve in the presence of an aberrant subclavian artery (B).

**Figure 4:**
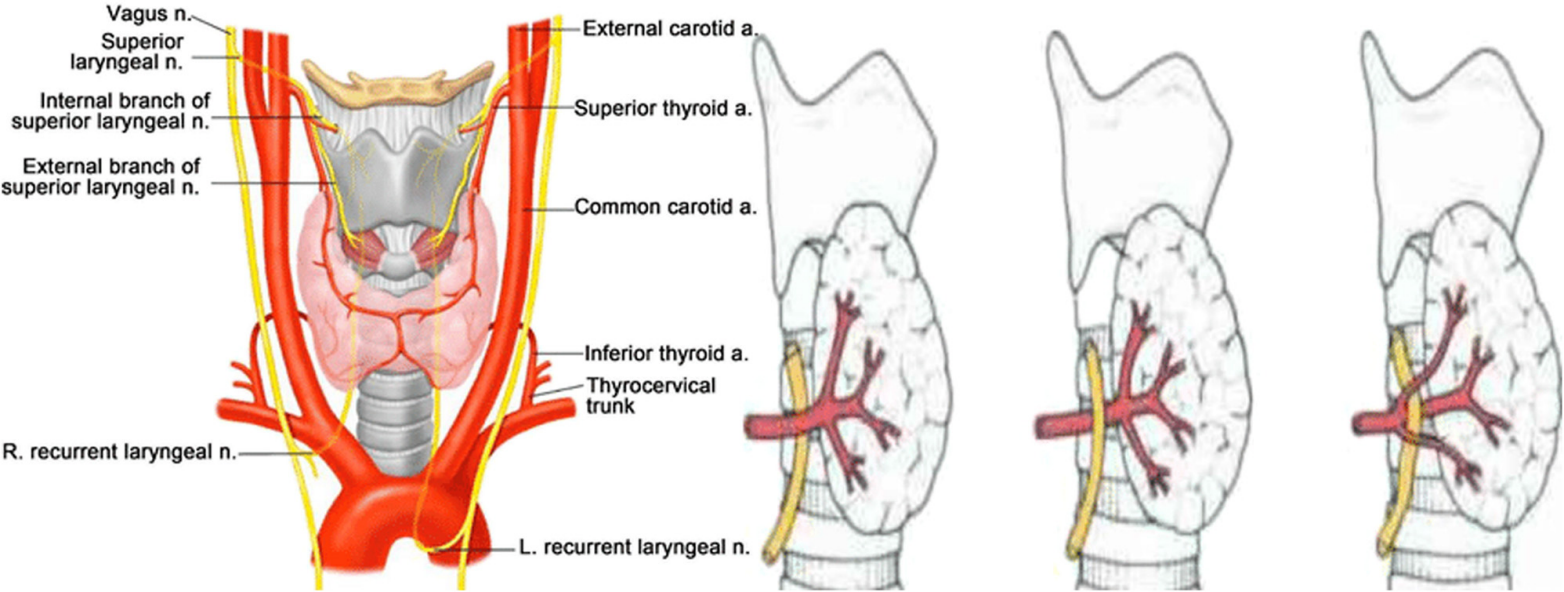
Anatomy of the recurrent laryngeal nerve and its relationship of the inferior thyroid artery and recurrent laryngeal nerve.

**Figure 5:**
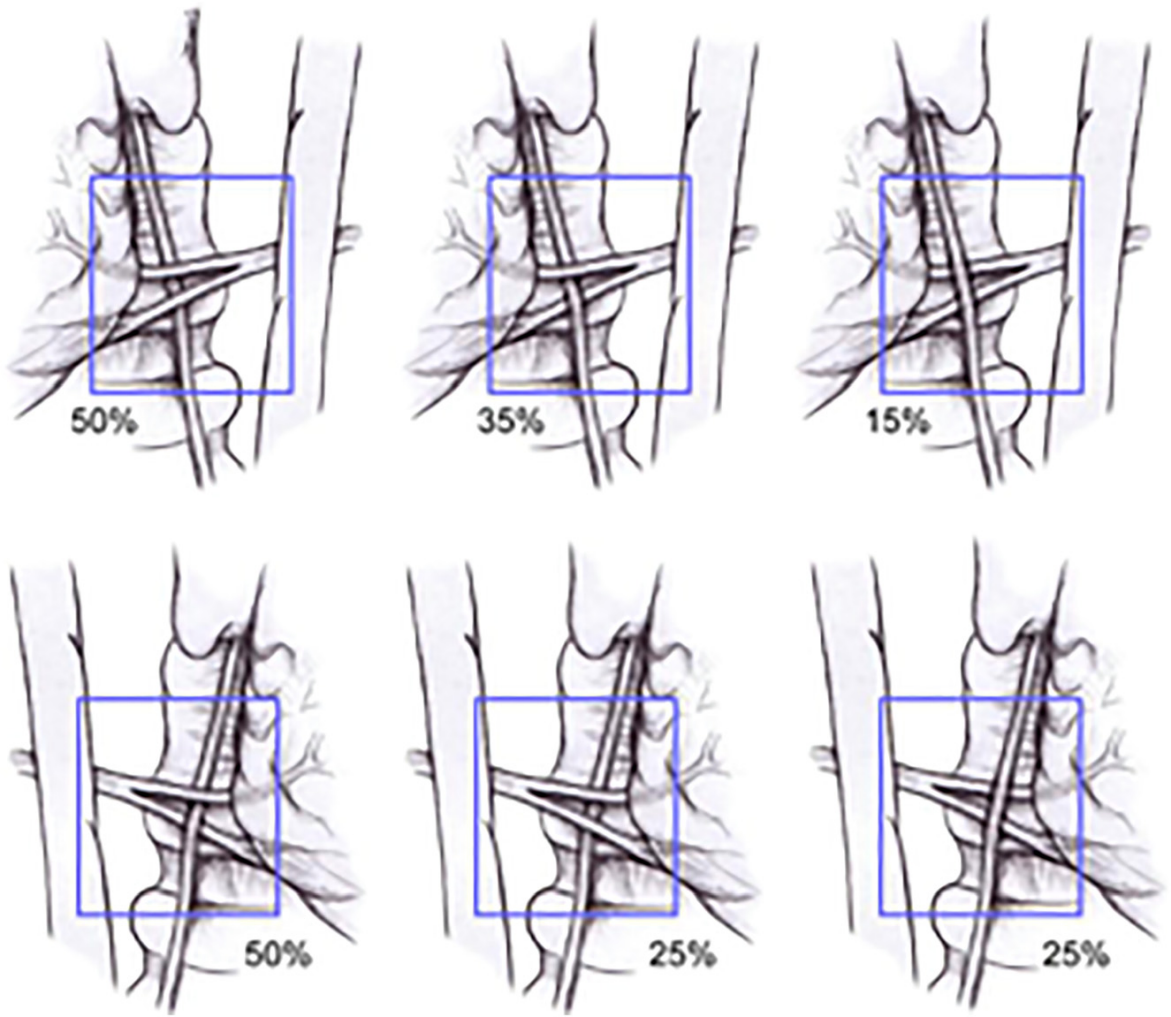
Relationship of the recurrent laryngeal nerve (RLN) to the left and right inferior thyroid arteries.

It is of utmost importance to note that a non-recurrent laryngeal nerve or direct branch of the vagus may also be identified on the right side in around 1% of cases [33]. A nonRLN occurs when a retro oesophageal right subclavian artery arises from the dorsal side of the aortic arch, and the branches from the vagus at the cricoid cartilage level enter the larynx without looping around the subclavian artery (Figure 3). This is not an issue on the left side, where the nonrecurrent laryngeal nerve has been rarely reported in patients [31]. The anatomic relationship among the RLN and the inferior thyroid artery branches is also not a reliable landmark for identifying the nerve [34, 35]. Usually, the nerve courses between the two branches, but it is not advisable to use the inferior thyroid artery as an anatomic landmark to identify the recurrent laryngeal nerve due to this unpredictability (Figure 4). On the right side, the nerve runs between the artery branches in 50% of patients, anterior to the artery in 25% and posterior in 25%. On the left side, the nerve runs posteriorly to the artery in 50% of patients and between the branches in approximately 35% (Figure 5).

The operative risk is associated with the extension of the resection, the type of surgery, the volume of the gland and the occurrence of thyroid disease (Graves’ disease, cancers etc.) [29, 36]. Common mechanisms of injury include complete or partial transection, traction, contusion, crush, burn, misplaced ligature and compromised blood supply. The nerve is less likely to be damaged if directly visualized during at least a portion of the operation. Medial retraction of the thyroid lobe remains a matter of preparation, practice, and choice of the surgeon, whether the nerve is exposed caudally to the inferior thyroid artery early in operation or after some mobilization of the superior and inferior poles [18, 29, 30, 37].

In most cases, irrespective of its previous caudal detection, the distal course of the nerve (2–3 cm), where the damage is most likely to occur, can not be seen until this stage in the operation. In the first month after surgery, Bergenfelz et al. reported 142 unilateral recurrent nerve paralyses, but nerve damage was only visualized in 14 cases during surgery [18].

The damage to the recurrent laryngeal nerve may be transient or permanent, unilateral or bilateral. The unilateral lesion causes dysphonia due to the immobility of the ipsilateral vocal cord and can be followed by difficulties with wheezing and liquid swallowing. An unusual complication is the bilateral lesion, associated with extreme episodes of breathlessness (0.4 percent) [36].

In the preoperative period, laryngoscopic examination is helpful to recognize damage and dysfunction already present, especially if the patient has undergone previous surgery in the cervical region. According to Echternach, who conducted pre- and post-operative laryngoscopy, 20% of laryngeal dysfunction patients had prior alterations in the vocal cords [37].

In the field of technique and instrumentation, considerable progress has been made. Ultrasound or radiofrequency systems can be replaced or supplemented by traditional methods (knot and tie ligation, metal clips and electrocoagulation), but the use of these devices has not demonstrated substantial changes in the incidence of nerve injury [38, 39]. When inserting hemostatic sutures, great care should be taken, so they are not positioned deep enough to encompass the nerve. We defined the nerve in this area for teaching purposes over the last decade, even though we were sure that the nerve was lateral or posterior to Berry’s ligament being transected, and did not sustain a permanent nerve injury. In cases in which the distal nerve was not already exposed after mobilizing the Zuckerkandl tubercle, this technique usually takes no more than a few seconds to do [31].

During thyroidectomy, nerve monitoring is a valuable tool for recognizing the nerve and recording its vitality. Intraoperative monitoring of RLN has allowed a reduction of injury rates of the recurrent nerve [40]. Barczynski et al. reported that the frequency of transient recurrent laryngeal nerve paresis was decreased by intraoperative neuromonitoring (2.9 percent for high-risk patients and 0.9 percent for low-risk patients) [41]. Interestingly, a meta-analysis by Higgins et al. found that systematic visualization of the RLN was still the best technique to prevent nerve injury, using the nerve stimulator with an RLN injury rate of 3.25 percent vs. 3.12 percent for nerve visualization [42].

Treatment for recurrent laryngeal nerve damage should be symptom-based. If there is dyspnea after extubation, reintubation is recommended to restore ventilation. To decrease laryngeal eodema, high-dose corticosteroid therapy is given for 48 h. Corticotherapy is followed by wide-spectrum antibiotic coverage and PPI treatment, which tends to reduce the risk of developing granuloma of the larynx. Persistent vocal cord immobility and severe dyspnea require the performance of a tracheostomy. Vocal cord mobility recovery was observed as late as 12 months after surgery. When vocal cord paralysis is in adduction, arytenoidectomy with a posterior vocal cord resection that restores space for air passage and respiratory function is a possible surgical remediation [43]. Laser posterior cordectomy, which has been known to be an excellent and safe treatment for upper airway obstruction relief, may also be used [44].

For unilateral RLN palsy and vocal cord paralysis during adduction, the tolerance of the patient and the impact of RLN palsy on the quality of life, respiratory function and phonation must first be assessed [31]. Remedial surgical techniques are medialization of the vocal cord by intracordal injection of autologous material when vocal cord paralysis is in abduction, thyroplasty consisting of implantation into the paralyzed vocal cord by thyrotomy of different materials and adduction of the arytenoid muscle [29, 45]. While it has been proposed to reconstruct the recurrent laryngeal nerve when nerve damage is recognized during surgery, the results are uncertain [46]. Finally, voice re-education is recommended for patients with recurrent laryngeal nerve palsy.

#### Superior laryngeal nerve injury

The external branch of the superior laryngeal nerve (SLN) is a branch of the vagus nerve (X cranial nerve) and is the most commonly injured nerve in thyroid surgery, with an injury rate of 0–25%.

**Figure 6:**
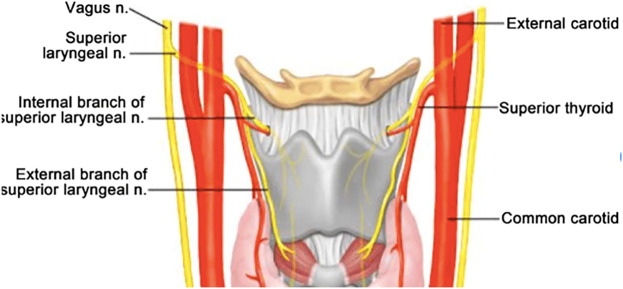
Anatomy of the superior laryngeal nerve.

The SLN has an internal and an external branch (Figure 6). The internal one provides sensory innervation to the larynx and enters it through the thyrohyoid membrane, which means it should not be at risk during thyroidectomy. The external branch has an anatomical relationship with the superior thyroid vessels, and before it ends at the cricothyroid muscle, it moves inferiorly along the inferior constrictor lateral surface. The cricothyroid muscle is involved in elongation of the vocal folds and trauma to this nerve results in the inability to lengthen the vocal folds and leads to high-pitched sound [47].

The external branch of the SLN is involved with the branches of the superior thyroid artery in a critical area 1.5–2 cm from the thyroid capsule. A recent study found that more than 1 cm above the upper pole of the thyroid gland, the nerve crosses the superior thyroid artery at 42%, less than 1 cm above the upper pole at 30% or under the upper pole at 14% [47].

It is widely accepted that for preventing the SLN, ligation of the terminal branches of the superior thyroid artery as close as possible to the thyroid capsule is recommended. Electrophysiologic monitoring of the SLN is not recommended [48]. If the SLN is injured, the only available treatment is speech therapy [49].

#### Thyrotoxic storm

Thyrotoxic storm or thyrotoxic crisis is a life-threatening condition that leads to excessive release of thyroid hormones in patients with thyrotoxicosis. It is an unusual complication that may result from manipulation of the thyroid gland in patients with hyperthyroidism. It can occur preoperatively, during the surgery, or postoperatively and can be prevented by preoperative management with antithyroid drugs [50]. The primary symptom of thyrotoxic storm is hyperpyrexia due to importantly high metabolism. The patient develops sinus tachycardia, hypertension and high-output cardiac failure, irritability and restlessness and thyrotoxicosis can also progress to alteration of mental status (delirium, seizures and coma). Gastrointestinal manifestations can also be evident and include diarrhoea, vomiting, jaundice and abdominal pain.

If thyrotoxic crisis occurs during thyroid surgery, the surgeon should stop the procedure and β-blockers, propylthiouracil, sodium iodine and steroids should be administrated intravenously. The use of cooling blankets and cooled intravenous fluids are essential for reducing the patient’s high body temperature. American Thyroid Association guidelines recommend that patients undergoing thyroidectomy should be managed preoperatively with methimazole and potassium iodide (KI). There is also the recommendation to render patients euthyroid with antithyroid medication is an effort to reduce the risk of thyroid storm that could be precipitated by the stress of surgery. KI is recommended to reduce thyroid gland vascularity, to improve operative visualization and to reduce operative complications [50].

## Conclusions

In conclusion, complications following thyroidectomy are predictable but not always preventable and require a prolongation of hospitalization, resulting in higher patient management costs. Surely, the occurrence of these complications can be minimized by attentive operator action during surgical procedures. In order to sustain the complication rate at very low levels, the surgeon must have a complete anatomical understanding of not only the normal anatomy of the central visceral compartment of the neck, but also of the common variations of the laryngeal nerves and parathyroid glands. It is clear that surgeons should be professionally qualified to do all of this, obey best practice, and retain their level of dedication and good communication until the result is ensured. The patient is not only fulfilled in this way, but adequately relieved of the issue.

## Supplementary Material

Supplementary Material

## References

[1] Gourin CG, Tufano RP, Forastiere AA, Koch WM, Pawlik TM, Bristow RE (2010). Volume-based trends in thyroid surgery. Arch Otolaryngol Head Neck Surg.

[2] Mansberger AR (1988). One hundred years of surgical management of hyperthyroidism. Ann Surg.

[3] Cernea CR, Brandão LG (2008). Kocher e a histo’ria da tireoidectomia–Rev. Bras Cir Cab Pesc. ..

[4] Landerholm K, Wasner AM, J€arhult J (2014). Incidence and risk factors for injuries to the recurrent laryngeal nerve during neck surgery in the moderate-volume setting. Langenbeck’s Arch Surg.

[5] Sosa JA, Bowman HM, Tielsch JM, Powe NR, Gordon TA, Udelsman R (1998). The importance of surgeon experience for clinical and economic outcomes from thyroidectomy. Ann Surg.

[6] Minuto MN, Reina S, Monti E, Ansaldo GL, Varaldo E (2019). Morbidity following thyroid surgery: acceptable rates and how to manage complicated patients. J Endocrinol Invest.

[7] Almquist M, Hallgrimsson P, Nordenström E, Bergenfelz A (2014). Prediction of permanent hypoparathyroidism after total thyroidectomy. World J Surg.

[8] Cho JN, Park WS, Min SY (2016). Predictors and risk factors of hypoparathyroidism after total thyroidectomy. Int J Surg.

[9] McIntyre RC, Eisenach JH, Pearlman NW, Ridgeway CE, Liechty RD (1997). Intrathyroidal parathyroid glands can be a cause of failed cervical exploration for hyperparathyroidism. Am J Surg.

[10] Libutti SK, Bartlett DL, Jaskowiak NT, Skarulis M, Marx SJ, Spiegel AM (1997). The role of thyroid resection during reoperation for persistent or recurrent hyperparathyroidism. Surgery.

[11] Cernea CR, Brandão LG, Hojaij FC, Carlucci DD, Montenegro FLM, Plopper C (2010). How to minimize complications in thyroid surgery?. Auris Nasus Larynx.

[12] Dedivitis RA, Aires FT, Cernea CR (2017). Hypoparathyroidism after thyroidectomy: prevention, assessment and management. Curr Opin Otolaryngol Head Neck Surg.

[13] Herranz Gonza´ lez-Botas J, Lourido Piedrahita D (2013). Hypocalcaemia after total thyroidectomy: incidence, control and treatment. Acta Otorrinolaringol Esp.

[14] Song CM, Jung JH, Ji YB, Min HJ, Ahn YH, Tae K (2014). Relationship between hypoparathyroidism and the number of parathyroid glands preserved during thyroidectomy. World J Surg Oncol.

[15] Khan MI, Waguespack SG, Hu MI (2011). Medical management of postsurgical hypoparathyroidism. Endocr Pract.

[16] Kakava K, Tournis S, Papadakis G, Karelas I, Stampouloglou P, Kassi E (2016). Postsurgical hypoparathyroidism: a systematic review. Vivo.

[17] Park I, Rhu J, Woo JW, Choi JH, Kim JS, Kim JH (2016). Preserving parathyroid gland vasculature to reduce postthyroidectomy hypocalcaemia. World J Surg.

[18] Bergenfelz A, Jansson S, Kristoffersson A, Mårtensson H, Reihnér E, Wallin G (2008). Complications to thyroid surgery: results as reported in a database from a multicenter audit comprising 3,660 patients. Langenbeck’s Arch Surg.

[19] Ronga G, Fragasso G, Fiorentino A, Paserio E, Todino V, Tummarello MA (1988). Prevalence of parathyroid insufficiency after thyroidectomy: study of 1037 cases. Ital J Surg Sci.

[20] Al-Dhahri SF, Mubasher M, Mufarji K, Allam OS, Terkawi AS (2014). Factors predicting post- thyroidectomy hypoparathyroidism recovery. World J Surg.

[21] Godballe C, Madsen AR, Pedersen HB, Sørensen CH, Pedersen U, Frisch T (2009). Post-thyroidectomyhemorrhage: a national study of patients treated at the Danish departments of ENT Head and Neck Surgery. Eur Arch Oto-Rhino-Laryngol.

[22] Promberger R, Ott J, Kober F, Seeman R, Freissmuth M, Hermann M (2012). Risk factors for postoperativebleeding after thyroid surgery. Br J Surg.

[23] Oltmann S, Alhefdhi A, Rajaei M, Schneider D, Sippel R, Chen H (2016). Antiplatelet and anticoagulant medications significantly increase the risk of postoperative hematoma: review of over 4500 thyroid and parathyroid procedures. Ann Surg Oncol.

[24] Lo Bianco S, Cavallaro D, Okatyeva V, Buffone A, Cannizzaro MA (2015). Thyroidectomy: natural drainage or negative drainage? Experience with randomized single-center study. Ann Ital Chir.

[25] Cannizzaro MA, Lo Bianco S, Borzı` L, Cavallaro A, Buffone A (2014). The use of FOCUS Harmonic scalpel compared to conventional haemostasis (knot and tie ligation) for thyroid surgery: a prospective randomized study. SpringerPlus.

[26] Gourin CG, Johnson FT, Randolph GW (2003). Surgery of the thyroid, parathyroid, glands. Postoperative complications.

[27] Smith RB, Coughlin A (2016). Thyroidectomy hemostasis. OtolaryngolClin North Am.

[28] Pironi D, Pontone S, Vendettuoli M, Podzemny V, Mascagni D, Arcieri S (2014). Prevention of complications during reoperative thyroid surgery. Clin Ter.

[29] Hartl DM, Travagli JP, Leboulleux S, Baudin E, Brasnu DF, Schlumberger M (2005). Current concepts in the management of unilate’ral recurrent laryngeal nerve paralysis after thyroid surgery. J Clin Endocrinol Metab.

[30] Echtemach M, Maurer C, Mencke T, Schilling M, Verse T, Richter B (2009). Laryngeal complications after thyroidectomy. Arch Surg.

[31] Tom Reeve, Norman W, Thompson (2000). Complications of thyroid surgery: how to avoid them, how to manage them and observations on their possible effect on the whole patient. World J Sure.

[32] Chan WF, Lo CY (2006). Pitfalls of intraoperative neuromonitoring for predicting postoperative recurrent laryngeal nerve function during thyroidectomy. World J Surg.

[33] Hunt PS, Poole M, Reeve TS (1968). A reappraisal of the surgical anatomy of the thyroid and parathyroid glands. Br J Surg.

[34] Lahey F (1941). AIDS in avoiding serious complications in thyroidectomy. Ann Surg.

[35] Lahey F (1948). Anatomy of the recurrent nerve. Ann Surg.

[36] Rosato L, Avenia N, Bernante P, Palma MD, Gulino G, Nasi PG (2004). Complications of thyroid surgery: analysis of a multicentric study on 14,934 patients operated on in Italy over 5 years. World J Surg.

[37] Yao HS, Wang Q, Wang WJ, Ruan CP (2009). Prospective clinical trials of thyroidectomy with LigaSure vs. conventional vessel ligation: a systematic review and meta-analysis. Arch Surg.

[38] Ecker T, Carvalho AL, Choe JH, Walosek G, Preuss KJ (2010). Hemostasis in thyroid surgery: harmonic scalpel versus other techniques: a meta-analysis. Otolaryngol Head Neck Surg.

[39] Lahey F (1938). Routine dissection and demonstration of recurrent laryngeal nerve in subtotal thyroidectomy. Surg Gynecol Obstet.

[40] Anuwong A, Lavazza M, Kim HY, Wu CW, Rausei S, Pappalardo V (2016). Recurrent laryngeal nerve management in thyroid surgery: consequences of routine visualization, application of intermittent, standardized and continuous nerve monitoring. Updates Surg.

[41] Barczynski M, Konturek A, Cichon S (2009). Randomized clinical trial of visualization versus neuromonitoring of recurrent laryngeal nerves during thyroidectomy. Br J Surg.

[42] Higgins TS, Gupta R, Ketcham AS, Sataloff RT, Wadsworth JT, Sinacori JT (2011). Recurrent laryngeal nerve monitoring versus identification alone on post-thyroidectomy true vocal fold palsy: a meta-analysis. Laryngoscope.

[43] Dispenza F, Dispenza C, Marchese D, Kulamarva G, Saraniti C (2012). Treatment of bilateral vocal cord paralysis following permanent recurrent laryngeal nerve injury. Am J Otolaryngology Head and Neck Med Surg.

[44] Mawaddah A, Marina M, Halimuddin S, Razif M, Abdullah S (2016). A ten-year Kuala Lumpur review on laser posterior cordectomy for bilateral vocal fold immobility. Malays J Med Sci.

[45] Baujat B, Delbove H, Wagner I, Fugain C, de Corbie`re S, Chabolle F (2001). Laryngeal immobility following thyroid surgery. Ann Chir.

[46] Miyauchi A, Inoue H, Tomoda C, Fukushima M, Kihara M, Higashiyama T (2009). Improvement in phonation after reconstruction of the recurrent laryngeal nerve in patients with thyroid cancer invading the nerve. Surgery.

[47] Cernea CR, Ferraz AR, Nishio S, Dutra A, Hojaij FC, dos Santos LR (1992). Surgical anatomy of the external branch of the superior laryngeal nerve. Head Neck.

[48] Cernea CR, Ferraz AR, Furlani J, Monteiro S, Nishio S, Hojaij FC (1992). Identification of the external branch of the superior laryngeal nerve during thyroidectomy. Am J Surg.

[49] Chandrasekhar SS, Randolph GW, Seidman MD, Rosenfeld RM, Angelos P, Barkmeier-Kraemer J (2013). Clinical practice guideline: improving voice outcomes after thyroid surgery. Otolaryngol Head Neck Surg.

[50] Ross DS, Burch HB, Cooper DS, Greenlee MC, Laurberg P, Maia AL (2016). 2016 American Thyroid Association guidelines for diagnosis and management of hyperthyroidism and other causes of thyrotoxicosis. Thyroid.

