# Supplementary material for: Surgical tips and techniques to avoid complications of thyroid surgery
Source: Innov Surg Sci. 2022 Oct 11;7(3-4):115–23. doi: 10.1515/iss-2021-0038 (PMC9742273; doi:10.1515/iss-2021-0038)
Supplement: Supplementary file 1 — Supplementary Material [file j_iss-2021-0038_suppl.pdf]

Reviewer Assessment

C. Stefanou et al.: Surgical Tips and Techniques to avoid Complications of Thyroid Surgery

Reviewers' Comments to Original Submission

Reviewer 1: anonymous

Date received: 13-Jun-2022
Reviewer recommendation: **Return to author for minor modifications**
Reviewer overall scoring: **High**

Assessment Form scores: 5 = High/Yes; 3 = Medium/Adequate; 1 = Low

Is the subject area appropriate for the journal	5				
Does the title clearly reflect the paper's content?	5				
Does the abstract clearly reflect the paper's content	5				
Do the keywords clearly reflect the paper's content?	5				
Does the introduction present the problem clearly?		4			
Are the results/ conclusions justified?	5				
How comprehensive and up-to-date is the subject matter presented?	5				
How adequate is the data presentation?		4			
Are units and terminology used correctly?		4			
Is the number of cases adequate?		4			
Are the experimental methods/ clinical studies adequate?			3		
Is the length appropriate in relation to the content?	5				
Does the reader get new insights from the article?		4			
Please rate the practical significance.	5				
Please rate the accuracy of methods.		4			
Please rate the statistical evaluation and quality control.		4			
Please rate the appropriateness of the figures and tables.	5				
Please rate the appropriateness of the references.		4			
Please evaluate the writing style and use of language.	5				
Please judge the overall scientific quality of the manuscript.			3		
Are the methods used worthy of reproduction in greater deal?	No				
Would you be willing to review a revision of this manuscript?	Yes				

Comments to author: Good paper. Just one thing, in the abstract you mentioned the method: literature, but in the main part, the method is missing. I would appreciate to know how many and which literature was seen.

Reviewer 2: Geffcken, Christine

Date received: 10-Jul-2022
Reviewer recommendation: **Accept in present form**
Reviewer overall scoring: **High**

Assessment Form scores: 5 = High/Yes; 3 = Medium/Adequate; 1 = Low

Is the subject area appropriate for the journal	5				
Does the title clearly reflect the paper's content?	5				
Does the abstract clearly reflect the paper's content	5				
Do the keywords clearly reflect the paper's content?	5				
Does the introduction present the problem clearly?	5				
Are the results/ conclusions justified?		4			
How comprehensive and up-to-date is the subject matter presented?		4			
How adequate is the data presentation?		4			
Are units and terminology used correctly?		4			
Is the number of cases adequate?		4			
Are the experimental methods/ clinical studies adequate?		4			
Is the length appropriate in relation to the content?	5				
Does the reader get new insights from the article?			3		
Please rate the practical significance.		4			
Please rate the accuracy of methods.			3		
Please rate the statistical evaluation and quality control.			3		
Please rate the appropriateness of the figures and tables.		4			
Please rate the appropriateness of the references.		4			
Please evaluate the writing style and use of language.		4			
Please judge the overall scientific quality of the manuscript.		4			
Are the methods used worthy of reproduction in greater deal?					
Would you be willing to review a revision of this manuscript?	Yes				

Comments to author: Good overview.

Authors' Response to Reviewer Comments

Date received: 11-Jul-2022

Response to reviewer 1

Line 25 (page 1)
Methods: MEDLINE database (PubMed) platform was used as a search engine and the articles related to the topic were selected using the keywords combination "thyroid surgery and complications ".

Line 25 (page 2)
Complications of Thyroid Surgery and how to avoid them

Comments by the Editor-in-Chief to Revised Submission

All reviewer comments were addressed adequately and the manuscript should be published in its present stage.
